# Effects of Sea Buckthorn Flavonoids on Growth Performance, Blood Biochemical Indexes, Rumen Fermentation and Rumen Bacteria of Hu Sheep Fed with High-Concentrate Diet

**DOI:** 10.3390/ani16152403

**Published:** 2026-08-04

**Authors:** Junlong Zhang, Caixia Shi, Wenguang Zhang, Risu Na, Mingjuan Gu, Lin Zhu, Jie Wang, Haoyuan Tian, Xin Li, Jing Jin, Liye Xiong, Tiankai Wang, Shuhan Zhao

**Affiliations:** 1 College of Animal Science, Inner Mongolia Agricultural University, Hohhot 010018, China; zhangjunlong1018@163.com (J.Z.); narsanjargal@imau.edu.cn (R.N.); gmj0119@yeah.net (M.G.); zhulinynacxhs@163.com (L.Z.); 15075621281@163.com (H.T.); lx_chst@163.com (X.L.); 18586101355@163.com (J.J.); xiongliye@outlook.com (L.X.); m15848295526@163.com (T.W.); zhaoshuhan0478@126.com (S.Z.); 2 Inner Mongolia Autonomous Region Engineering Research Center of Agricultural Genomic Big Data, Hohhot 010018, China; atcgnmbi@aliyun.com; 3 Key Laboratory of Animal Nutrition and Feed Science of Inner Mongolia Higher Education Institutions, Hohhot 010018, China; 4 China-Mongolia Biomacromolecule Application ‘Belt and Road’ Joint Laboratory, Hohhot 010018, China; 5 Bayannur Institute of Agriculture and Animal Husbandry, Bayannur 015000, China; 15174867475@163.com

**Keywords:** sea buckthorn flavonoids, high-concentrate diet, Hu sheep, growth performance, rumen bacteria

## Abstract

This study evaluated the effects of dietary sea buckthorn flavonoid (SF) supplementation on growth performance, serum biochemical, antioxidant, and immune indices, rumen fermentation, and ruminal bacterial composition in Hu sheep fed a high-concentrate diet. Thirty 6-month-old fattening Hu sheep were randomly assigned to a high-concentrate control diet (HC) or the same diet supplemented with 0.30% SF (HT; *n* = 15 per group) for 45 days. Compared with the HC group, SF supplementation increased average daily gain (*p* = 0.04) and reduced the feed conversion ratio (*p* = 0.01) without affecting average daily feed intake. Serum total protein, alanine aminotransferase, total antioxidant capacity, IgA, IgG, IL-2, IL-8, and IL-1β were increased in the HT group (*p* ≤ 0.05). Ruminal acetate and valerate concentrations increased (*p* = 0.01 and *p* = 0.03, respectively), and total volatile fatty acid concentration showed an increasing tendency (*p* = 0.06), whereas rumen pH was unaffected. The relative abundances of *Succiniclasticum* and *Acidaminococcus* increased in the HT group (*p* < 0.05), indicating modulation of specific ruminal bacterial taxa. In conclusion, supplementation with 0.30% SF improved growth efficiency, antioxidant status, selected immune indices, and specific rumen fermentation and bacterial responses in Hu sheep fed a high-concentrate diet. However, the isolated increase in serum alanine aminotransferase may indicate hepatic metabolic adaptation or mild liver stress. Dose–response and time-course studies incorporating liver-specific biomarkers and histopathological evaluation are required to clarify the hepatic safety of sea buckthorn flavonoid supplementation.

## Simple Summary

High-concentrate diets are widely used to improve growth efficiency in intensive sheep production, but prolonged feeding may adversely affect rumen fermentation and animal health. Sea buckthorn flavonoids are plant-derived bioactive compounds with potential antioxidant and immunomodulatory effects. In this study, 30 6-month-old fattening male Hu sheep were randomly assigned to a high-concentrate diet group (no supplement or 0.30% sea buckthorn flavonoids) for 45 days. Supplementation increased average daily gain and improved feed conversion without affecting feed intake. It also increased serum total antioxidant capacity and several immune-related indicators, increased ruminal acetate and valerate concentrations, and altered the relative abundance of specific rumen bacterial genera. Total volatile fatty acid concentration showed an increasing tendency, whereas rumen pH remained unchanged. Serum alanine aminotransferase activity also increased, and its biological significance requires further investigation. These findings suggest that sea buckthorn flavonoids may improve growth efficiency and selected physiological and ruminal responses in Hu sheep fed a high-concentrate diet. Further dose–response studies are needed to determine the optimal supplementation level.

## 1. Introduction

High-concentrate diets are widely used in intensive sheep production because they increase dietary energy density and support rapid growth [1]. However, these diets provide large amounts of rapidly fermentable carbohydrates. These substrates are rapidly fermented by rumen microorganisms, increasing the production and accumulation of volatile fatty acids and, under certain conditions, lactate. When acid production exceeds the buffering and absorptive capacity of the rumen, ruminal pH declines and the risk of subacute ruminal acidosis increases. Persistent low ruminal pH can suppress acid-sensitive microorganisms, disrupt ruminal microbial homeostasis, and promote the release of lipopolysaccharide and histamine, thereby contributing to ruminal epithelial inflammation, liver abscesses, and metabolic dysfunction [2,3]. Therefore, nutritional strategies that maintain production efficiency while reducing the adverse effects of high-concentrate feeding are needed.

Plant-derived polyphenols have attracted attention as feed additives because of their antioxidant, anti-inflammatory, antimicrobial, and immunomodulatory properties. In an in vitro model of subacute ruminal acidosis, pomegranate peel polyphenols reduced histamine concentrations and altered volatile fatty acid production [4]. In sheep, dietary supplementation with 0.25% sea buckthorn flavonoids affected carcass traits, serum protein metabolism, enzyme activity, and cytokine secretion [5,6]. Sea buckthorn flavonoids (SF) are bioactive polyphenolic compounds with documented antioxidant [7], anti-inflammatory [8], and immune-related activities [9,10]. These findings indicate that SF may have value in ruminant nutrition; however, previous studies have generally examined isolated outcomes rather than integrating growth performance, systemic biochemical responses, ruminal fermentation, and ruminal bacterial composition under high-concentrate feeding conditions.

The effects of dietary SF on these interconnected responses in high-concentrate-fed Hu sheep (Ovis aries) remain insufficiently characterized. We hypothesized that SF supplementation would improve growth performance and feed efficiency while enhancing serum antioxidant and immune status and modulating ruminal fermentation and bacterial composition. Accordingly, this study evaluated growth performance; serum biochemical, antioxidant, and immune indices; ruminal fermentation parameters; and ruminal bacterial composition in fattening Hu sheep receiving a high-concentrate diet with or without SF. The findings provide evidence for evaluating SF as a plant-derived feed additive in intensive sheep production.

## 2. Materials and Methods

### 2.1. Animals and Experimental Design

This study was approved by the Ethics Committee of Inner Mongolia Agricultural University (NND2023090). A completely randomized grouping design was used in the experiment. Thirty 6-month-old fattening male Hu sheep with similar body weight and good health were selected. According to the principle of no significant difference in body weight, they were divided into two groups, 15 in each group. The control group (HC group) was fed a basal high-concentrate diet; the experimental group (HT group) was supplemented with 0.30% sea buckthorn flavonoids (3 g/kg diet) in the basal diet. The sea buckthorn flavonoids used in the test were powder, with a purity of 30%, and were provided by Inner Mongolia Astronaut Sand Industry Co., Ltd., Hohhot, Inner Mongolia, China.

Before feeding, sea buckthorn flavonoids were pre-mixed with a small amount of concentrate and then mixed into the whole diet for feeding; the control group was fed the same amount of basal diet without additional sea buckthorn flavonoids. The raw material composition and nutritional level of the basic diet are shown in Table 1. In this experiment, the whole concentrate feeding mode (no hay feeding) was used to simulate the production scene of high-concentrate feeding. All sheep were raised separately in a well-ventilated 2 m × 2 m (4 m^2^/only) indoor enclosure. Each barn is equipped with a separate feeder and water dispenser. Feed twice a day at 08:00 and 17:00, free access to food and water. The enclosure was cleaned and disinfected regularly and naturally ventilated.

Before the start of the experiment, all experimental sheep were clinically examined to confirm that they were in good mental state, had normal feeding, and had no digestive tract disease or parasitic infection; the deworming and basic immunization have been completed in a unified manner. The pre-feeding period was 15 days, and the formal test period was 45 days. This experiment was carried out in the experimental pasture of the College of Animal Science, Inner Mongolia Agricultural University, and its feeding management and operation process were in line with the ethical norms of animal experiments.

### 2.2. Sample Collection

On the penultimate day of the experiment, the jugular vein blood was taken before morning feeding by vacuum blood collection (China Liuyang Sanli Medical Technology Development Co., Ltd., Liuyang, China), centrifuged for 15 min by 3500 r/min low-temperature centrifuge, and the serum was taken and packed in 1.5 mL sterile centrifuge tube (Hangzhou Bori Technology Co., Ltd., Hangzhou, China), frozen at −20 °C. On the last day of the experiment, 40 mL of rumen fluid was collected using a rumen sampler (Shandong Huachen Medical Device Co., Ltd., Weifang, China) immediately after morning feeding (0 h) and at 3 and 6 h thereafter. After the test, the test sheep were humanely euthanized. The rumen contents of 9 Hu sheep in each group were collected for 16S rRNA sequencing of rumen bacteria. The collected rumen samples were placed in a 5 mL cryopreservation tube (Wuxi Ness Biotechnology Co., Ltd., Wuxi, China) and frozen in −80 °C dry ice.

### 2.3. Index Determination Method

#### 2.3.1. Determination of Growth Performance

The experimental sheep were weighed on an empty stomach before morning feeding on the 1st and 45th day of the formal period, and the initial body weight (IW) and final body weight (FW) were recorded, and the average daily gain during the trial period was calculated (ADG = total weight gain/number of days in the trial period). At the same time, the daily feed intake and residual feed intake were recorded to calculate the average daily feed intake (ADFI = (feed intake-residual feed intake)/trial days). On this basis, the feed-to-weight ratio (FCR = ADFI/ADG) was calculated.

#### 2.3.2. Determination of Serum Indicators

All the indexes in serum were detected by a Hitachi 3110 automatic biochemical analyzer (Shanghai Hitachi Diagnostic Products Co., Ltd., Shanghai, China) and a microplate reader (Leikang Hengtai Trading Co., Ltd., Beijing, China). Total protein (TP), albumin (ALB), globulin (GLO), aspartate aminotransferase (AST), alanine aminotransferase (ALT), alkaline phosphatase (ALP) and total bilirubin (TBIL) were measured. Catalase (CAT), total antioxidant capacity (TAC), malondialdehyde (MDA), superoxide dismutase (SOD); immunoglobulin M (IgM), immunoglobulin A (IgA), immunoglobulin G (IgG), interleukin-2 (IL-2), interleukin-6 (IL-6), interleukin-8 (IL-8), interleukin-1β (IL-1β) and tumor necrosis factor-α (TNF-α) All antioxidant and immune indexes were detected using commercial assay kits (Nanjing Jiancheng Bioengineering Institute, Nanjing, China), and all operations were carried out in strict accordance with the kit instructions.

#### 2.3.3. Determination of Rumen Fermentation Parameters

The pH value was measured immediately after sampling using a portable pH meter; the concentration of NH3-N was fixed with 0.20 mol/L hydrochloric acid and detected by colorimetry [11]. The concentration of VFA was determined by the method of Li Min [12] after being fixed with 25% metaphosphoric acid.

#### 2.3.4. 16 S rRNA Detection

In this experiment, the CTAB/SDS method was used to extract total genomic DNA from rumen samples. The concentration and purity of DNA were detected by 1% agar electrophoresis, and sterile water was diluted to 1 ng/μL during this process. The diluted genomic DNA was used as a template, and the test area was selected. The PCR amplification program was performed using Phusion^®^ High-Fidelity PCR Master Mix GC Buffer, specific primers, and high-efficiency enzymes from New England Biolabs, Ipswich, MA, USA. The cycle conditions included first denaturation at 98 °C for 1 min, then 30 cycles at 98 °C (10 s), 50 °C (30 s), and 72 °C (30 s), and finally extension at 72 °C for 5 min. Firstly, the PCR product was detected by 2% agarose gel, and then the target band was recovered using the adhesive kit. Finally, the sequencing adapter was connected to both sides of the amplified DNA fragment by DNA adhesive. The DNA fragments were purified and selected by AMpure PB magnetic beads, and the SMRT Bell library was constructed. The purified fragments were redissolved with buffer, and then the fragments of specific size were screened with BluePipin fragments, and then the DNA fragments were purified by AMpure PB magnetic beads. The constructed library was quantified by Qubit concentration, and the fragment size was detected by Agilent2100 (Agilent Technologies, Santa Clara, CA, USA). Finally, the PacBio platform was used for sequencing. Subsequently, the sequencing results were analyzed in depth by Alpha diversity analysis, Beta diversity analysis, and species difference analysis, aiming to comprehensively explore the composition characteristics and community structure of microorganisms.

The 16S rRNA detection data were exported in the form of a BAM file. After CCS correction, filtering out too-long or too-short sequences, distinguishing sample data according to the barcode sequence, and finally performing primer removal, effective clean reads were obtained. The statistical data for each part of the data processing process is shown in Table 2. In this experiment, HT1.1–HT1.9 was the control group and HT2.1–HT3.9 was the experimental group.

### 2.4. Statistical Analysis

All data were analyzed using IBM SPSS Statistics 26 software after preliminary statistics by Excel. *p* < 0.05 was considered statistically significant, and *p* < 0.01 was considered extremely statistically significant. The final data results are represented by Mean ± SD.

## 3. Results

### 3.1. Growth Performance

It can be seen from Table 3 that compared with the HC group, the ADG of the HT group was significantly increased (*p* = 0.04). FCR was significantly decreased (*p* = 0.01), and IW, FW and ADFI were not significantly affected (*p* > 0.05).

### 3.2. Serum Biochemical Indicators

It can be seen from Table 4 that compared with the HC group, TP in the HT group was significantly increased (*p* = 0.05), and ALT was significantly increased (*p* = 0.01). ALB, GLO, A/G, AST, ALP, TBIL, CRE, UREA, GLU, CHOL, TG, Ca and *p* had no significant effect (*p* > 0.05).

### 3.3. Serum Antioxidant Levels and Immune Indicators

As shown in Table 5, compared with the HC group, the TAC of the HT group was significantly increased (*p* = 0.04). CAT, SOD and MDA had no significant effect (*p* > 0.05). It can be seen from Table 6 that IgA and IL-1β in the HT group were significantly increased (*p* = 0.01), and IgG, IL-2 and IL-8 were significantly increased (*p* < 0.05).

### 3.4. Rumen Fermentation Parameters

It can be seen from Table 7 that compared with the HC group, the rumen pH of the HT group had no significant effect at 0 h, 3 h and 6 h (*p* > 0.05). It can be seen from Table 8 that the acetic acid in the HT group increased significantly (*p* = 0.01), the valeric acid increased significantly (*p* = 0.03), and the T-VFA tended to increase (*p* = 0.06). NH_3_-N, propionic acid, isobutyric acid, butyric acid, isovaleric acid and ethylene-propylene ratio had no significant effect (*p* > 0.05).

### 3.5. Rumen Bacterial Flora

#### 3.5.1. Flora Distribution Analysis

**Figure 1:**
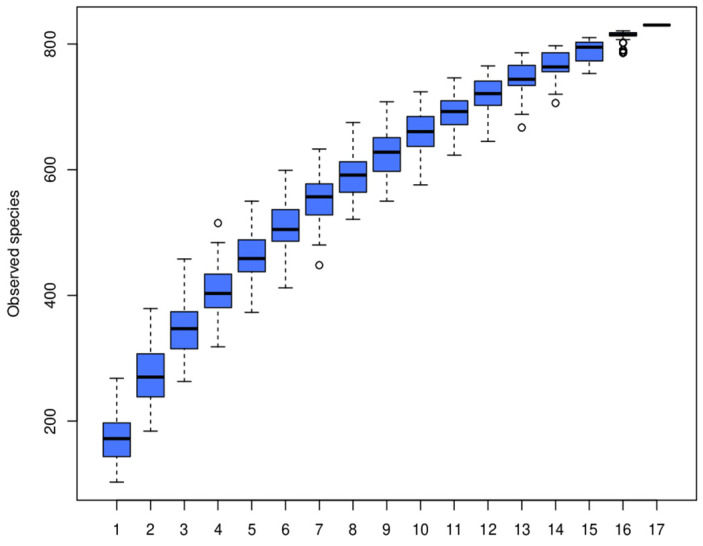
Species accumulation boxplot of rumen bacterial operational taxonomic units (OTUs) across the 18 samples. Circles represent outlier values.

**Figure 2:**
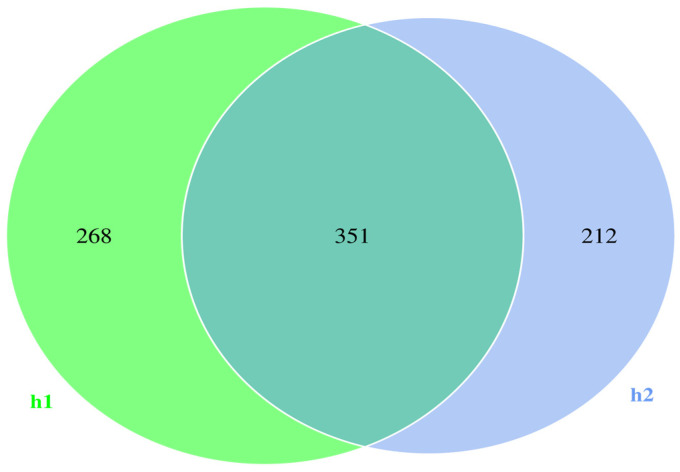
Venn diagram showing shared and unique rumen bacterial OTUs between the HC and HT groups. HC, high-concentrate diet; HT, high-concentrate diet supplemented with 0.30% sea buckthorn flavonoids.

In this study, the species accumulation box plot was drawn based on the operational taxonomic unit (OTU) level. The results showed that with the increase in the number of samples, the number of species gradually increased and tended to be stable, indicating that the sequencing depth was sufficient, and the sequencing coverage was considered adequate for describing the bacterial taxa detected in the available samples of the rumen bacterial community of Hu sheep, which could be used for subsequent analysis of bacterial diversity and structural differences (Figure 1). Further comparison of rumen bacteria between the two groups by Venn diagram showed that there were 351 OTUs in the two groups. Among them, there were 268 unique OTUs in the HC group and 212 unique OTUs in the HT group (Figure 2).

#### 3.5.2. Sample Complexity Analysis

**Figure 3:**
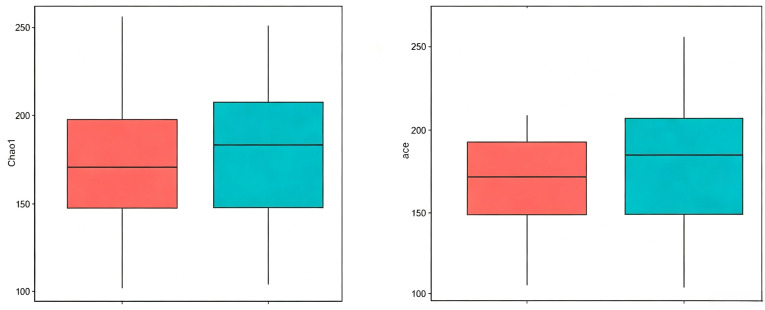
Alpha diversity of rumen bacterial communities in the HC and HT groups, assessed using the Chao1 (**left**) and ACE (**right**) richness indices. Red boxes represent the HC group, cyan boxes represent the HT group. HC, high-concentrate diet; HT, high-concentrate diet supplemented with 0.30% sea buckthorn flavonoids.

**Figure 4:**
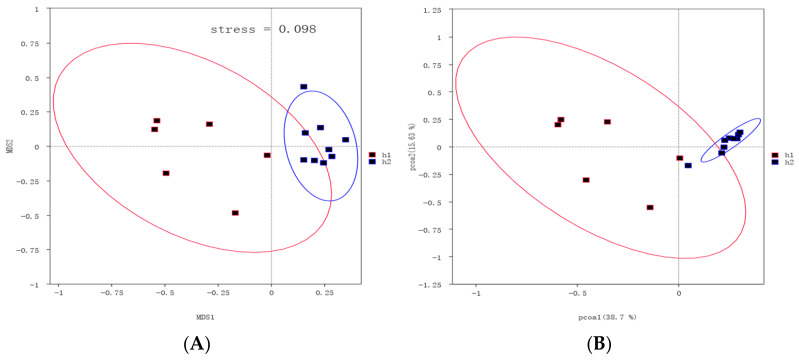
Beta-diversity ordination of rumen bacterial communities in the HC and HT groups: (**A**) non-metric multidimensional scaling; (**B**) principal coordinate analysis (PCoA). Ellipses indicate the distribution of samples within each group.

In this study, Alpha diversity analysis was performed on two groups of samples. The results of Alpha diversity showed that compared with the HC group, the Chao1 index and ACE index of the HT group showed an increasing trend, indicating that the species richness of the rumen bacterial community of Hu sheep increased after the addition of sea buckthorn flavonoids (Figure 3). The results of PCoA and NMDS analysis showed that the bacterial community structure of the two groups exhibited a certain separation trend. Among them, the stress value from the NMDS analysis is 0.098, indicating that the sorting results have good reliability. The results of PCoA analysis further showed that there were differences in the distribution areas of the two groups of samples, indicating that the addition of sea buckthorn flavonoids to the high-concentrate diet changed the rumen bacterial community structure of Hu sheep to a certain extent (Figure 4).

#### 3.5.3. Analysis of Bacterial Community Structure

In this study, the taxonomic composition of the flora structure of the two groups of samples was analyzed at the phylum, family and genus levels, and the dominant flora with higher relative abundance was screened for comparison.

**Figure 5:**
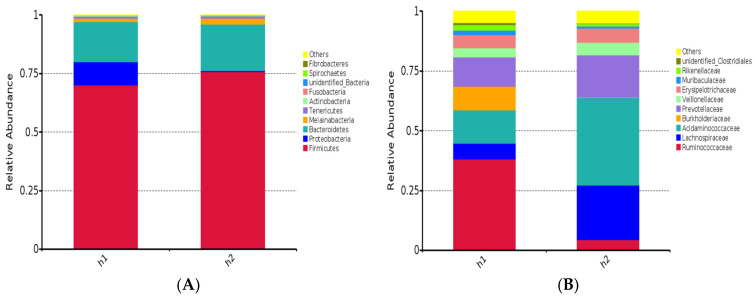
Relative abundance of dominant rumen bacterial taxa in the HC and HT groups at the (**A**) phylum and (**B**) family levels. Taxa with low relative abundance are combined as “Others”.

At the phylum level. The rumen bacteria of the Hu sheep were mainly composed of *Firmicutes*, *Proteobacteria*, *Bacteroidota*, *Melainabacteria*, *Tenericutes*, *Actinobacteria*, *Fusobacteria*, unidentified_*Bacteria*, *Spirochaetes* and *Fibrobacteres*. Among them, *Firmicutes*, *Proteobacteria* and *Bacteroidota* were the main dominant phyla in the rumen of the two groups of Hu sheep (Figure 5A).

At the family level. The rumen bacteria of Hu sheep mainly include *Ruminococcaceae*, *Lachnospiraceae*, *Acidaminococcaceae*, *Burkholderiaceae*, *Prevotellaceae*, *Veillonellaceae*, *Erysipelotrichaceae*, *Muribaculaceae*, *Rikenellaceae* and unidentified_*Clostridiales*. The composition of the main fungi in different treatment groups was similar, but the relative abundance of some fungi was different (Figure 5B).

**Figure 6:**
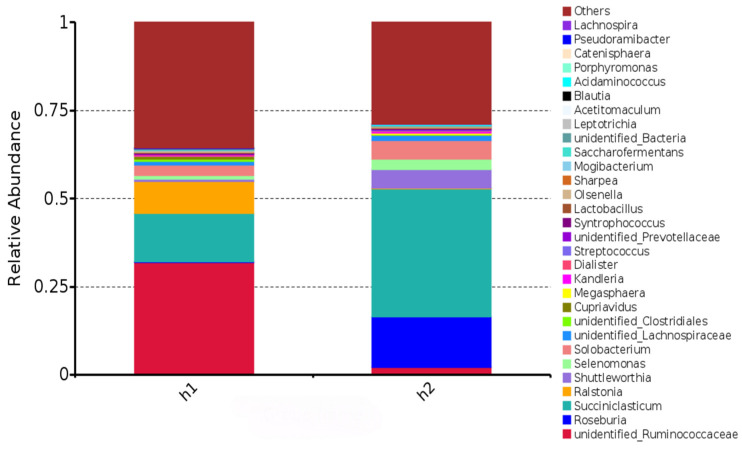
Relative abundance of the 30 most abundant rumen bacterial genera in the HC and HT groups. Genera with lower relative abundance are combined as “Others”.

At the genus level, this study selected the top 30 genera of relative abundance for comparison. The results show that the main bacteria in the rumen of the two groups of Hu sheep included unidentified_*Ruminococcaceae*, *Roseburia*, *Succiniclasticum*, *Ralstonia, Shuttleworthia*, *Selenomonas*, *Solobacterium*, unidentified_*Lachnospiraceae*, unidentified_*Clostridiales*, *Cupriavidus*, *Megasphaera*, *Kandleria* and *Dialister* (Figure 6). Compared with the HC group, the relative abundance of some genera in the HT group changed, among which the relative abundance of *Succiniclasticum*, *Roseburia*, *Solobacterium* and *Shuttleworthia* increased, while the relative abundance of unidentified_*Ruminococcaceae* decreased.

**Figure 7:**
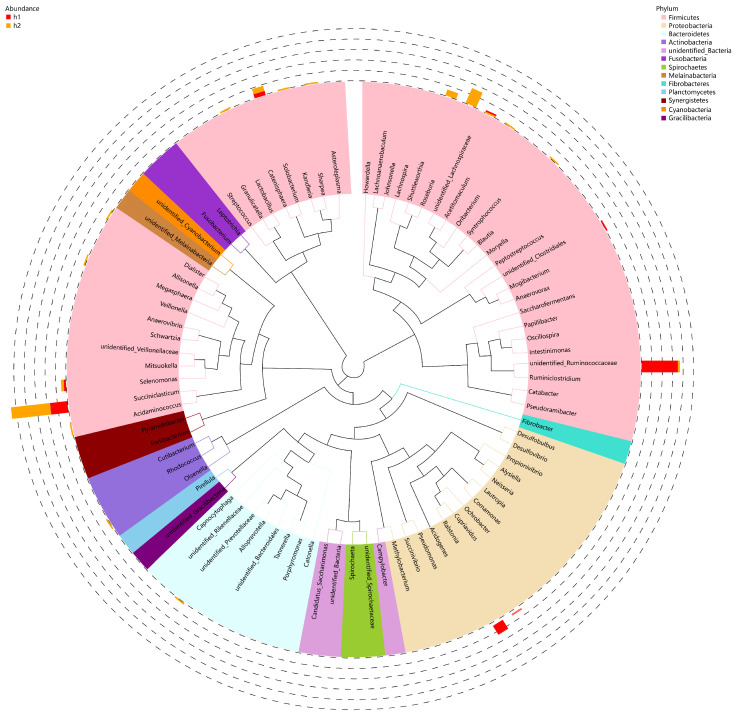
Phylogenetic tree of the 100 most abundant rumen bacterial genera in the HC and HT groups. Branch background colors indicate phylum-level classification, and outer bars indicate relative abundance in each group.

In addition, in order to explore the phylogenetic relationship between the dominant bacteria at the genus level, this study performed multiple sequence alignments based on the representative sequences of the top 100 genera with high relative abundance and constructed a phylogenetic tree. The results showed that different genera had certain clustering characteristics in phylogenetic relationships, indicating that the rumen flora of Hu sheep had a more complex compositional structure at the genus level. Combined with the results of relative abundance analysis at the genus level, it can be seen that the addition of sea buckthorn flavonoids may change the composition ratio of some dominant bacteria in the rumen of Hu sheep to a certain extent (Figure 7).

#### 3.5.4. Analysis of Differential Flora

**Figure 8:**
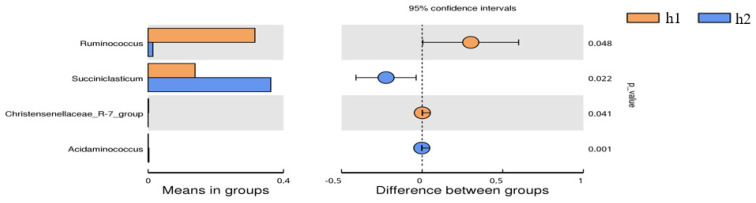
Genus-level differential abundance analysis between the HC and HT groups using an independent-samples *t*-test. Points and horizontal lines show the between-group difference and 95% confidence interval; *p*-values are shown on the right.

**Figure 9:**
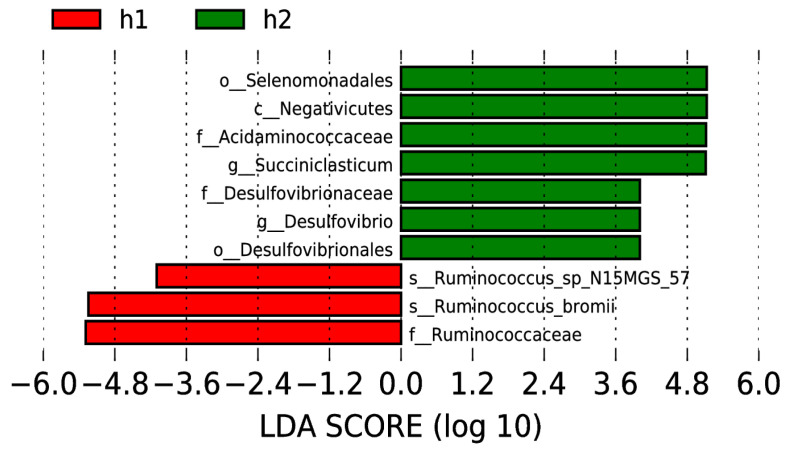
Linear discriminant analysis effect size (LEfSe) scores of differentially abundant rumen bacterial taxa between the HC and HT groups. Taxa with an LDA score > 4 are displayed.

**Figure 10:**
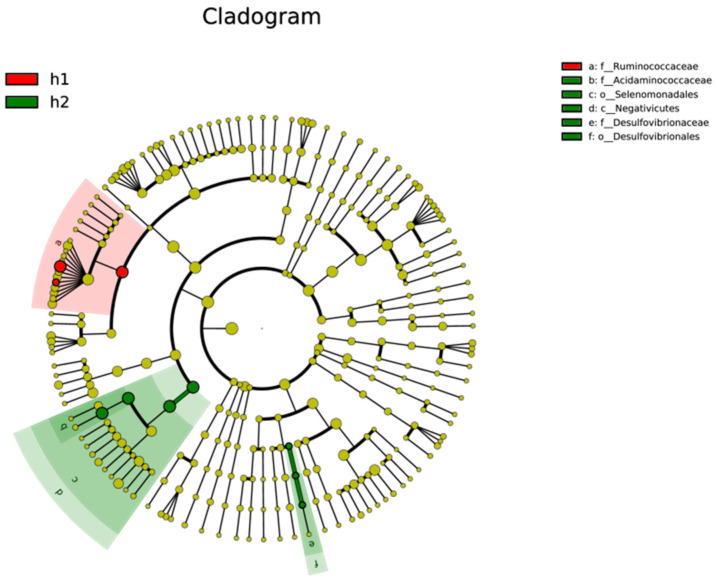
LEfSe cladogram showing the phylogenetic distribution of differentially abundant rumen bacterial taxa between the HC and HT groups. Red and green nodes indicate taxa enriched in h1 and h2, respectively; yellow nodes indicate taxa without significant differences.

In order to further clarify the effect of sea buckthorn flavonoids on the difference in rumen bacterial community in Hu sheep, *t*-test and LEfSE (LDA effect size) analyses were used to compare the two groups of samples. At the genus level, *t*-test analysis was performed with *p* < 0.05 as the screening criteria. The results showed that the relative abundance of *Ruminococcus*, *Succiniclasticum*, *Christensenellaceae_R-7_group* and *Acidaminococcus* was significantly different between the two groups (*p* < 0.05, Figure 8). LEfSE analysis was further used to screen the differential marker bacteria between groups, LDA > 4 was used as the criterion for differential flora, and the evolutionary branch diagram of differential flora was constructed. The results showed that in the HC group, a total of 1 family level and 2 species levels of significantly different flora were detected. Among them, the differential flora at the family level was *Ruminococcaceae*; *ruminococcus*_sp_N15MGS_57 and *Ruminococcus_bromii* were included in the differential flora at the species level. In the HT group, a total of 1 class level, 2 order levels, 2 family levels and 2 genus levels were detected. Among them, the differential flora at the family level was *Ruminococcaceae*; *ruminococcus*_sp_N15MGS_57 and *Ruminococcus_bromii* were included in the differential flora at the species level. In the HT group, a total of 1 class level, 2 order levels, 2 family levels and 2 genus levels were detected. Among them, the class-level differential flora was anaerobic bacteria (*Negativicutes*); the order-level differential flora were *Selenomonadales* and *Desulfovibrionales*; the differential flora at the family level were *Acidaminococcaceae* and *Desulfovibrionaceae*. The differential flora at the genus level were *Succiniclasticum* and *Desulfovibrio* (Figure 9 and Figure 10).

#### 3.5.5. Correlation Analysis Between Rumen Bacteria and Growth Performance

**Figure 11:**
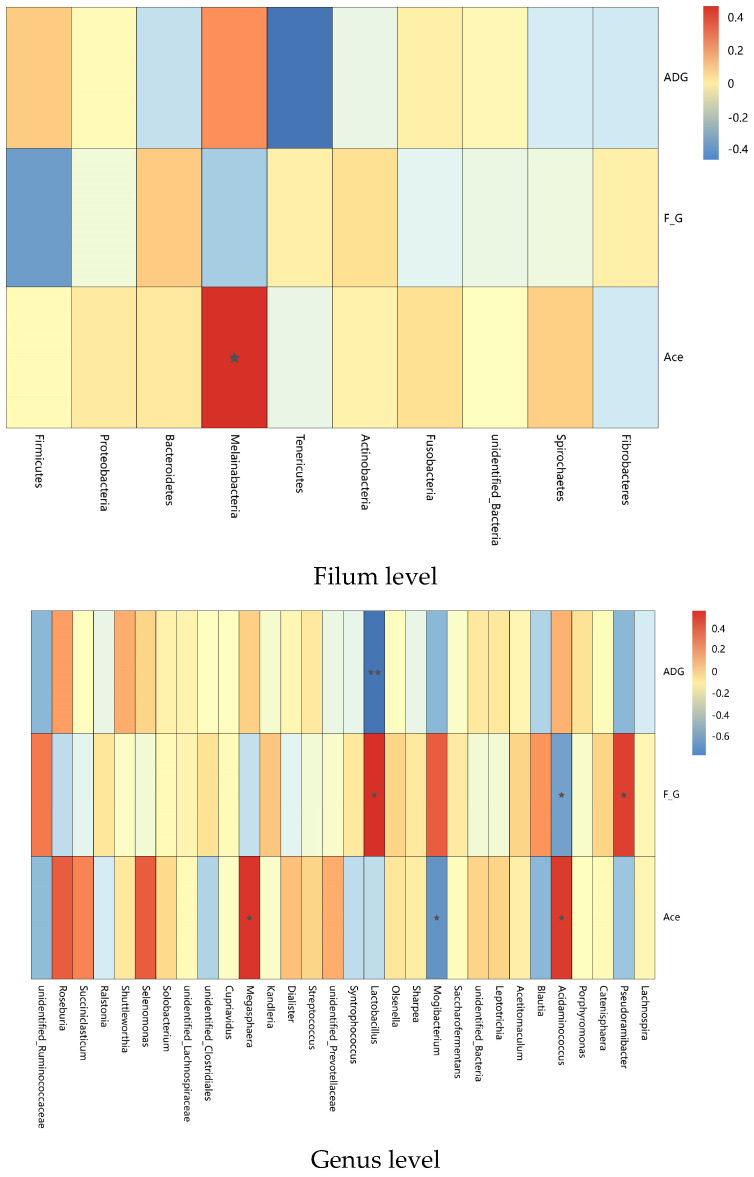
Spearman correlation heatmaps between rumen bacterial taxa and growth and fermentation variables at the phylum level (**upper panel**) and genus level (**lower panel**). ADG, average daily gain; F/G, feed-to-gain ratio; Ace, ruminal acetate concentration. * *p* < 0.05; ** *p* < 0.01.

In order to explore the relationship between rumen bacterial community and growth performance and related phenotypic indexes of Hu sheep, the Spearman correlation analysis method was used to analyze the correlation between rumen bacteria at different classification levels and average daily gain, feed-to-weight ratio and rumen fermentation-related indexes. According to Figure 11, at the genus level, *Pseudoramibacter*, *Acidaminococcus* and *Lactobacillus* were significantly correlated with the ratio of feed to gain (*p* < 0.05). Among them, *Lactobacillus* was also significantly correlated with average daily gain (*p* < 0.05).

In terms of rumen fermentation-related indicators, the results of phylum-level analysis showed that there was a significant correlation between *Melainabacteria* and acetic acid content (*p* < 0.05). At the genus level, *Megasphaera*, *Acidaminococcus* and *Mogibacterium* were significantly correlated with acetic acid content (*p* < 0.05).

#### 3.5.6. Correlation Analysis Between Rumen Bacteria and Immune Indexes

**Figure 12:**
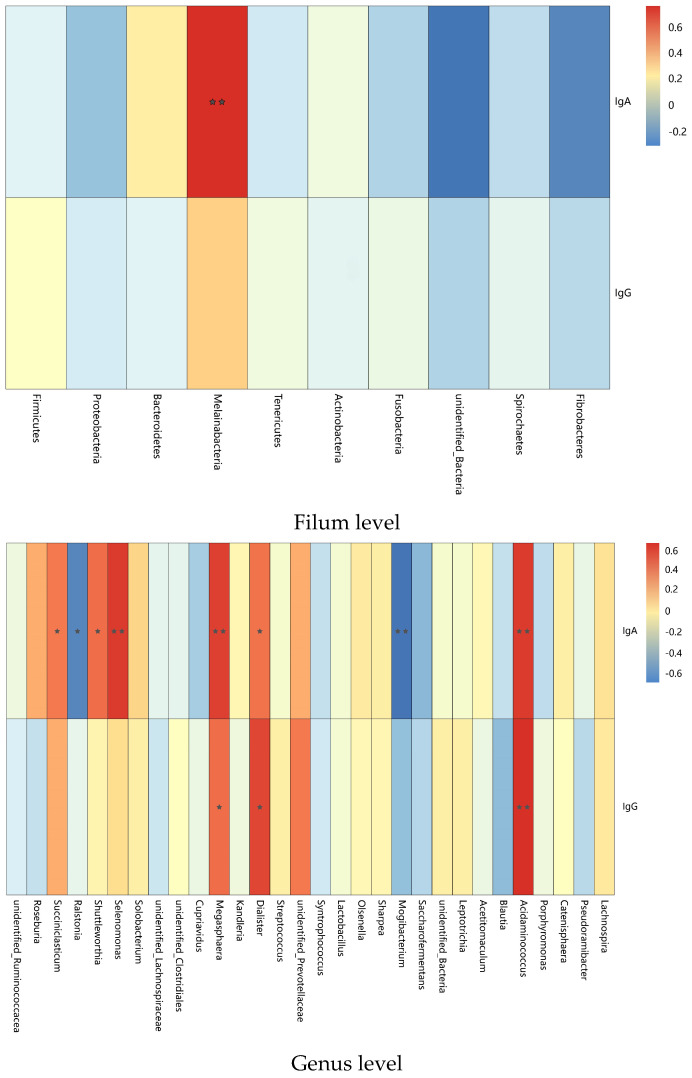
Spearman correlation heatmaps between rumen bacterial taxa and serum immunoglobulin indices at the phylum level (**upper panel**) and genus level (**lower panel**). IgA, immunoglobulin A; IgG, immunoglobulin G. * *p* < 0.05; ** *p* < 0.01.

In order to further explore the relationship between rumen bacterial community and immune function of Hu sheep, this study continued to use the Spearman correlation analysis method to analyze the correlation between rumen bacteria and serum immune indexes at different classification levels. It can be seen from Figure 12 that at the phylum level, *Melainabacteria* was significantly correlated with IgA (*p* < 0.05). At the genus level, *Acidaminococcus* was significantly correlated with IgG (*p* < 0.01), and *Dialister* and *Kandleria* were significantly correlated with IgG (*p* < 0.05). In addition, *Acidaminococcus*, *Megasphaera*, *Mogibacterium* and *Selenomonas* were significantly correlated with IgA (*p* < 0.01). *Dialister*, *Shuttleworthia*, *Succiniclasticum* and *Ralstonia* were significantly correlated with IgA (*p* < 0.05).

## 4. Discussion

Sea buckthorn flavonoids are a mixture composed of multiple plant polyphenols rather than a single compound. Dietary supplementation with 0.30% sea buckthorn flavonoids increased average daily gain and reduced the feed conversion ratio without significantly affecting average daily feed intake (Table 3), which is consistent with previous studies reporting the growth-promoting effects of sea buckthorn flavonoids in other livestock species [13,14]. These findings suggest that the improvement in feed efficiency resulted from an overall enhancement in nutrient utilization rather than an increase in feed intake. At the phylum level, *Firmicutes* and *Bacteroidota* were dominant in both groups, although descriptive differences in their relative abundances were observed (Figure 5A). Previous studies have demonstrated that flavonoid supplementation is associated with alterations in gut microbial composition [15]. Both dominant phyla contain microorganisms involved in carbohydrate degradation and amino acid metabolism [16], including taxa capable of degrading complex plant polymers [17,18].

Short-chain fatty acids produced through ruminal microbial fermentation are major energy substrates for ruminants [19]. In the present study, ruminal acetate and valerate concentrations increased, whereas total volatile fatty acids showed only an increasing trend (*p* = 0.06); ruminal pH and the acetate-to-propionate ratio were not significantly affected (Table 7 and Table 8). These findings are generally consistent with previous reports showing that dietary flavonoids can modify ruminal fermentation characteristics [20,21]. Acetate is absorbed through the ruminal epithelium and converted into acetyl-CoA, which supports ATP production and lipid synthesis, whereas valerate can be metabolized through pathways that generate acetyl-CoA and propionyl-CoA. Therefore, the higher acetate and valerate concentrations may have increased energy availability. However, because metabolic flux and tissue utilization were not measured, a causal explanation for the observed increase in body weight gain cannot be established. At the genus level, *Succiniclasticum* is known for converting succinate into propionate [22], whereas *Selenomonas* can utilize lactate and participate in propionate-related fermentation [23]. The positive correlation between *Melainabacteria* and acetate concentration [24], together with the observed genus-level differences, provides a hypothetical basis for microbial cross-feeding. The 16S rRNA amplicon analysis provides information on the taxonomic composition and relative abundance of ruminal bacteria, but it does not directly measure microbial functional genes, gene expression, enzyme activity, metabolite production, or metabolic flux. Therefore, the proposed roles of *Succiniclasticum*, *Acidaminococcus*, *Melainabacteria*, and other taxa were inferred from previously reported characteristics of related microorganisms rather than directly demonstrated in the present animals. In addition, correlations between bacterial relative abundance and growth, fermentation, or immune variables do not establish causal relationships. Consequently, the microbiota findings should be interpreted as taxonomic associations and hypothesis-generating observations rather than evidence of specific microbial mechanisms. Metagenomic, metatranscriptomic, metabolomic, targeted quantitative PCR, and culture-based analyses would be required for functional validation.

Supplementation with sea buckthorn flavonoids elevated serum total protein concentrations, while blood urea nitrogen and creatinine levels remained unchanged (Table 4). This pattern of alterations is consistent with modified protein metabolism. Comparable effects of plant flavonoids on serum biochemical parameters have been documented in other animal trials [25]. Quercetin is capable of modulating protein digestion and absorption [26] as well as altering in vitro ruminal fermentation profiles and microbial community structure [27]; meanwhile, members of *Acidaminococcaceae* participate in amino acid metabolism [28]. Serum ALT activity was significantly increased in the HT group, whereas AST, ALP, and total bilirubin were not significantly affected. This isolated increase in ALT should not be regarded solely as physiological variation. Under the background of forage-free high-concentrate feeding, it may indicate altered hepatic metabolic activity, an adaptive response associated with the hepatic biotransformation of flavonoids, or mild hepatocellular stress. The absence of significant changes in AST, ALP, and total bilirubin does not support overt hepatocellular injury or cholestasis; however, it does not exclude an early or subclinical hepatic response. Because liver histopathology, liver-specific biomarkers such as GLDH and GGT, circulating lipopolysaccharide, bile acids, hepatic oxidative and inflammatory markers, and flavonoid metabolites were not evaluated, the present study cannot distinguish hepatic metabolic adaptation from mild liver stress. High-concentrate feeding may trigger endotoxin-induced oxidative stress in the liver [29], and this mild elevation may reflect physiological variation or shifts in hepatic metabolic activity. Total antioxidant capacity was significantly enhanced, with no remarkable changes observed in catalase, superoxide dismutase or malondialdehyde (Table 5). This finding may align with the non-enzymatic antioxidant properties of sea buckthorn polyphenols, such as free radical scavenging and metal ion chelation [30,31].

Immunoglobulins serve as central mediators of humoral immune defense [32]. Dietary sea buckthorn flavonoids increased serum IgA and IgG concentrations, alongside elevated levels of IL-2, IL-8 and IL-1β (Table 6). The significant increase in serum IL-1β requires cautious interpretation because IL-1β is a major pro-inflammatory cytokine rather than a direct indicator of improved immune function. Together with the increase in IL-8 and the numerical increases in IL-6 and TNF-α, the elevated IL-1β concentration may indicate systemic immune activation or a low-grade inflammatory response. Alternatively, it may represent transient immune signaling associated with dietary flavonoid supplementation. However, blood samples were collected at only one time point, and acute-phase proteins, leukocyte profiles, circulating lipopolysaccharide, inflammasome-related markers, and tissue inflammatory responses were not evaluated. Therefore, the present data cannot determine whether the cytokine changes represent beneficial immune priming or an adverse inflammatory response. Previous investigations have also reported immunomodulatory effects of sea buckthorn flavonoids and other botanical flavonoid extracts [33,34]. The relative abundance of *Acidaminococcus* was positively correlated with serum IgA and IgG contents (Figure 12). Microbially derived short-chain fatty acids regulate mucosal IgA responses, and acetate has been validated to activate G protein-coupled receptor 43 in other experimental systems [35,36]. Neither receptor activation nor the mediation of immunoglobulin responses by *Acidaminococcus* metabolites was verified in the present study. Similarly, the correlation between *Selenomonas* and IL-8 cannot confirm activation of the Toll-like receptor 4/nuclear factor κB (TLR4/NF-κB) signaling pathway. Although links between sea buckthorn flavonoids and this pathway have been established in other disease models [37], their underlying functional mechanism in Hu sheep remains hypothetical and requires direct molecular validation for confirmation.

## 5. Conclusions

In summary, the addition of 0.3% SF to the high-concentrate diet of Hu sheep can improve the antioxidant capacity and immunity of Hu sheep, regulate rumen fermentation and bacterial flora, increase the production of total volatile fatty acids in the rumen, and ultimately improve growth performance.

## Figures and Tables

**Table 1:** Dietary composition and nutritional level.

Raw Material Composition	Content (%)	Nutritional Level	Content (%)
Corn	50.00	Metabolizable energy (MJ/kg)	10.22
Cottonseed meal	8.00	Crude protein	11.58
DDGS medium fat	7.50	Crude fat	3.18
Sunflower powder	30.00	Neutral detergent fiber	31.00
Stone powder	2.00	Acid detergent fiber	16.60
Sodium bicarbonate	0.50	Ca	0.90
Salt	1.00	P	0.42
Premix	1.00		
Total	100		

Note: Premix provides trace elements per kilogram of diet: Cu 15 mg, Fe 55 mg, Mn 40 mg, Se 0.30 mg, I 0.50 mg, Co 0.20 mg; vitamins: VA12000 IU, VD4000 IU, VE400 IU. Except for metabolic energy, other nutritional levels were measured.

**Table 2:** Data preprocessing statistics and quality control.

#Sample_Name	Clean_Reads (#)	Base (nt)	AvgLen (nt)
HT1.1	10,985	15,944,271	1451
HT1.2	10,692	15,554,146	1454
HT1.3	12,538	18,278,015	1457
HT1.4	20,614	30,119,209	1461
HT1.5	12,520	18,312,508	1462
HT1.6	17,956	26,280,147	1463
HT1.7	15,964	23,328,167	1461
HT1.8	19,518	28,383,093	1454
HT1.9	12,816	18,778,448	1465
HT2.1	18,782	27,560,400	1467
HT2.2	16,650	24,397,486	1465
HT2.3	16,715	24,449,429	1462
HT2.4	11,049	16,110,087	1458
HT2.5	13,338	19,491,998	1461
HT2.6	12,900	18,835,294	1460
HT2.7	16,062	23,503,053	1463
HT2.8	19,436	28,409,974	1461
HT2.9	18,674	28,834,785	1457

**Table 3:** Effect of sea buckthorn flavonoids on growth performance of Hu sheep in high-concentrate diet.

Item	HC	HT	*p*-Value
IW (kg)	42.24 ± 2.68	42.74 ± 3.07	0.72
FW (kg)	49.59 ± 3.14	50.72 ± 3.59	0.49
ADG (kg/d)	0.16 ± 0.05	0.30 ± 0.06	0.04
ADFI (kg/d)	1.12 ± 0.10	1.16 ± 0.08	0.41
FCR	7.83 ± 0.62	6.41 ± 0.73	0.01

Note: IW, initial body weight; FW, final body weight; ADG, average daily gain; ADFI, average daily feed intake; FCR, feed conversion ratio.

**Table 4:** Effect of sea buckthorn flavonoids on serum biochemical indexes of Hu sheep in high-concentrate diet.

Item	HC	HT	*p*-Value
TP (g/L)	63.94 ± 4.80	73.38 ± 12.99	0.05
ALB (g/L)	34.69 ± 3.87	38.14 ± 5.95	0.57
GLO (g/L)	29.25 ± 2.56	35.24 ± 5.09	0.41
A/G	1.19 ± 0.15	1.08 ± 0.24	0.76
AST (U/L)	102.90 ± 20.64	99.77 ± 21.74	0.80
ALT (U/L)	11.69 ± 1.11	14.15 ± 1.25	0.01
ALP (U/L)	365.40 ± 52.37	364.11 ± 74.90	0.91
TBIL (μmol/L)	0.49 ± 0.19	0.63 ± 0.25	0.24
CRE (μmol/L)	55.61 ± 4.37	60.41 ± 8.15	0.23
UREA (mmol/L)	6.99 ± 1.23	7.59 ± 1.83	0.47
GLU (mmol/L)	3.61 ± 0.71	3.89 ± 0.89	0.50
CHOL (mmol/L)	1.87 ± 0.14	1.64 ± 0.35	0.14
TG (mmol/L)	0.29 ± 0.07	0.30 ± 0.05	0.81
Ca (mmol/L)	2.23 ± 0.26	2.23 ± 0.27	0.99
P (mmol/L)	2.80 ± 0.38	2.73 ± 0.43	0.74

Note: TP, total protein; ALB, albumin; GLO, globulin; A/G, albumin to globulin ratio; AST, aspartate aminotransferase; ALT, alanine aminotransferase; ALP, alkaline phosphatase; TBIL, total bilirubin; CRE, creatinine; UREA, blood urea nitrogen; GLU, glucose; CHOL, total cholesterol; TG, triglyceride; Ca, calcium; P, phosphorus.

**Table 5:** Effect of sea buckthorn flavonoids on serum antioxidant level of Hu sheep in high-concentrate diet.

Item	HC	HT	*p*-Value
CAT (U/mL)	11.44 ± 6.87	12.89 ± 5.20	0.68
SOD (U/mL)	187.40 ± 42.97	193.33 ± 32.57	0.77
MDA (nmol/mL)	1.32 ± 0.49	1.19 ± 0.26	0.52
TAC (mmol/mL)	0.59 ± 0.03	0.64 ± 0.01	0.04

Note: CAT, catalase; SOD, superoxide dismutase; MDA, malondialdehyde; TAC, total antioxidant capacity.

**Table 6:** Effect of sea buckthorn flavonoids on serum immunity of Hu sheep in high-concentrate diet.

Item	HC	HT	*p*-Value
IgM (μg/mL)	28.47 ± 5.70	30.45 ± 3.84	0.42
IgA (μg/mL)	11.92 ± 0.92	14.15 ± 1.79	0.01
IgG (μg/mL)	21.14 ± 2.82	24.49 ± 2.82	0.03
IL-2 (pg/mL)	180.59 ± 26.11	252.28 ± 82.64	0.02
IL-6 (pg/mL)	110.68 ± 6.84	129.30 ± 30.17	0.09
IL-8 (pg/mL)	103.22 ± 7.36	114.33 ± 8.75	0.03
IL-1β (pg/mL)	174.57 ± 27.13	236.76 ± 61.08	0.01
TNF-α (pg/mL)	178.52 ± 24.92	219.72 ± 61.36	0.08

Note: Ig, immunoglobulin; IL, interleukin; TNF-α, tumor necrosis factor-α.

**Table 7:** Effect of high-concentrate diet supplemented with sea buckthorn flavonoids on rumen pH of Hu sheep.

Time	HC	HT	*p*-Value
0 h	6.02 ± 0.35	5.99 ± 0.33	0.82
3 h	5.52 ± 0.33	5.34 ± 0.48	0.46
6 h	5.54 ± 0.31	5.57 ± 0.61	0.91

**Table 8:** Effects of sea buckthorn flavonoids on NH_3_-N and volatile fatty acids in rumen of Hu sheep fed high-concentrate diet.

Item	HC	HT	*p*-Value
NH_3_-N (mg/100 mL)	5.97 ± 0.90	5.91 ± 0.81	0.90
T-VFA (mmol/L)	72.66 ± 8.54	88.31 ± 4.39	0.06
Acetic acid (mmol/L)	34.59 ± 3.48	46.44 ± 1.99	0.01
Propionic acid (mmol/L)	29.17 ± 7.00	29.55 ± 4.47	0.92
Isobutyric acid (mmol/L)	0.23 ± 0.09	0.33 ± 0.10	0.09
Butyric acid (mmol/L)	7.12 ± 2.08	8.70 ± 3.25	0.37
Isovaleric acid (mmol/L)	0.22 ± 0.06	0.19 ± 0.04	0.44
Valeric acid (mmol/L)	1.48 ± 0.71	2.86 ± 1.15	0.03
A/P	1.26 ± 0.21	1.18 ± 0.27	0.68

Note: NH_3_-N, ammonia nitrogen; T-VFA, total volatile fatty acids; A/P, ratio of acetic acid to propionic acid.

## Data Availability

The data presented in this study can be obtained from the text.
